
JOURNAL INFORMATION
==============================
NLM Title Abbreviation: Behav Neurol
Journal ID: Behav Neurol
NLM Journal ID: 8914585
Journal ID: BN

Behavioural Neurology

ISSN: 0953-4180
EISSN: 1875-8584
Publisher: Wiley

ARTICLE INFORMATION
==============================
PMCID: PMC5434321
PMID: 21422547
DOI: 10.3233/BEN-2010-0287
Article ID: 252843
Article version: 1
Subjects: Research Article

The Prognostic Value of Neuropsychological Examination after SAH

Penninx J. F. 1 2
Visser-Meily J. M. A. 3
Passier P. E. C. A. 3
Rinkel G. J. E. 2
Post M. W. 3
Van Zandvoort M. J. E. 1 2
1 Psychological Laboratory Heimholtz Institute Utrecht University The Netherlands
2 Rudolf Magnus Institute of Neuroscience Department of Neurology and Neurosurgery University Medical Centre Utrecht Utrecht The Netherlands
3 Rudolf Magnus Institute of Neuroscience and Centre of Excellence for Rehabilitation Medicine University Medical Centre Utrecht and De Hoogstraat Utrecht The Netherlands
Print publication date: 2010
Electronic publication date: 2011 Mar 7
Volume: 23
Issue: 4
First page: 173
Last page: 175
Received 2011 Jan 31; Accepted 2011 Jan 31
Copyright: Copyright © 2010 Hindawi Publishing Corporation and the authors.
Copyright year: 2010
License: This is an open access article distributed under the Creative Commons Attribution License, which permits unrestricted use, distribution, and reproduction in any medium, provided the original work is properly cited.
License URL: https://creativecommons.org/licenses/by/3.0/

==============================
Behavioural Neurology 23 (2010) 173–175
DOI 10.3233/BEN-2010-0287
IOS Press

### The prognostic value of neuropsychological examination after SAH

J.F. Penninx[a,b], J.M.A. Visser-Meily[c], P.E.C.A. Passier[c], G.J.E. Rinkel[b], M.W. Post[c]
M.J.E. Van Zandvoort[a,b]
[a]*Psychological Laboratory, Heimholtz Institute, Utrecht University, The Netherlands*
[b]*Rudolf Magnus Institute of Neuroscience, Department of Neurology and Neurosurgery, University Medical Centre Utrecht, Utrecht, The Netherlands*
[c]*Rudolf Magnus Institute of Neuroscience and Centre of Excellence for Rehabilitation Medicine, University Medical Centre Utrecht and De Hoogstraat, Utrecht, The Netherlands*

### 1. Introduction

Cognitive impairments are well known consequences of subarachnoid hemorrhage (SAH) [7]. In 50–60 % of the SAH survivors mild to severe cognitive dysfunctions have been reported [5,10]. Moreover, from the SAH survivors with a good neurological outcome (no physical disabilities) 30% remain cognitive impaired in the long term [10].

Memory appeared most frequently impaired [3,6]. However, evidence for more diffuse deficits, such as reduced psychomotor speed and decreased sustained attention, have been reported [7]. A recent review underlines the heterogeneity of the cognitive sequels after SAH and points towards memory, executive functioning and language as most frequently impaired [1]. Therefore, a clear-cut profile of the cognitive sequels of SAH is still lacking, and so far no predictors for long term cognitive functioning have been reported. Nevertheless, the majority of SAH patients experience cognitive complaints of influence on their quality of life, stressing the need for more knowledge in this area to optimise care in the long term. The aims of the present study are (a) to describe the course of cognitive functioning at three months and 1 year post SAH and (b) to search for sensitive predictors for cognitive functioning in the long term.

### 2. Methods

### 2.1. SAH patients and procedure

At our SAH-outpatient clinic a concise cognitive examination is administered within 3 months after a SAH from a ruptured aneurysm (*baseline measurement*). For 53 randomly assigned patients an extended evaluation took place at 12 to 18 months post onset. Patient group included 42 women (79%), with mean age of 54.4 (11.8) range of 32 to 85. Data on demographics and clinical condition, assessed by means of the Prognosis on Admission of Aneurysmal Subarachnoid Hemorrhage (PAASH) scale, were obtained from the prospectively collected database of the UMC Utrecht. A highly similar control group was assembled as reference sample consisting of 62 subjects all seen twice for neuropsychological examination. Both baseline (9 tasks) and follow-up (16 tasks) examination covered six major cognitive domains consisting of verbal and nonverbal neuropsychological tasks: Verbal and Nonverbal Memory, Attention, Executive functioning, Visuoconstruction and Perception, and Speed of Processing.

### 2.2. Statistical analyses

The PAASH was dichotomized resulting in good (PAASH $\leqslant$ I) or reduced (PAASH $>$I) clinical condition. The raw test scores on the individual neuropsy-

ISSN 0953-4180/10/$27.50 © 2010 – IOS Press and the authors. All rights reserved

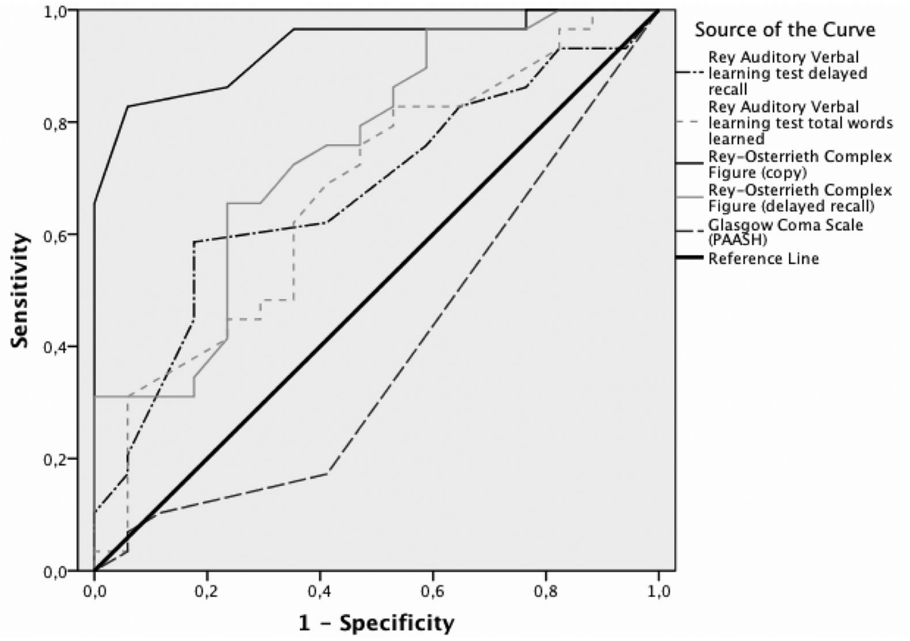

Fig. 1. ROC curve. Prognostic validity of neuropsychological tasks on cognitive functioning in the long term.

chological tasks were transformed into z-scores based on the reference sample. Subsequently, we averaged z-scores of individual tasks into domain scores. For classification of impairment, either at task or domain level, z-score $< -1.5$ was used. In addition, a sum score for overall cognitive integrity was composed by the mean z-score of all assessed tasks. This measure was dichotomised as z-score $> -0.5$ representing cognitively favourable outcome, and z-score $\leqslant -0.5$ as pointing toward the presence of cognitive problems that can hamper daily life [4].

Receiver-operator characteristic (ROC curves) were used to search for the best predictors for cognitive integrity in the long term amongst the neuropsychological tasks and the PAASH.

### 3. Results

At baseline, 20 of the 53 patients (38%) presented with a cognitive impairment in one or more domains. Deficits appeared as expected in a heterogeneous pattern with most deficits in the domains of verbal and nonverbal memory and visuoconstruction. Notably, in almost 1 out every 2 patients (45%) these impairments appeared persistent as compared to the same test battery at follow-up. Inspection of the recovery course showed that the domain Attention and Nonverbal memory demonstrated the largest recovery, whereas impairments in Visuoconstruction and Visuoperception remained over time.

ROC-analyses in search for sensitive predictors for long term cognitive functioning, represented as the dichotomised sum score of all tasks of the extended test battery, demonstrated that clinical condition (PAASH) had only limited non-significant predictive value; optimal cut-off PAASH: 1,5/6 with a corresponding sensitivity/specificity 17/59. The Rey-Osterrieth Complex Figure appeared to be the most sensitive predictor for long term cognitive functioning (AUC $= 0.93$; 95% CI: 0.86–1.00, $p < 0.0001$). The optimal cut-off score of 30,5/36 yielded a sensitivity/specificity rate of 96/65, indicative for a highly sensitive measurement [2].

### 4. Discussion

On the whole, our patients, showed both relatively good clinical and cognitive outcome in line with earlier findings. However, our study included selectively patients who visited outpatient clinics within three months post SAH, so these comprise the SAH survivors with expected good outcome. It is the more important to note that, the profile at baseline reflected a heterogeneous pattern of impairments across individuals and domains. This heterogeneity was in line with the literature [1]. Unexpected was that in almost half of the initially cognitive impaired patients (45%) deficits remained present over time, with impairments in visuoconstruction and perception as most persistent. Moreover, in search for a predictor for cognitive integrity in the long term, the Rey-Osterrieth complex figure copy emerged surprisingly as the most sensitive predictor.

Although the suggestion of this study could be that a SAH leads towards impairments in the visuoperception and construction, impairments are relatively mild and on group level do not resemble focal impairments in visual perception. The heterogeneity of the profile at baseline resembles the diffuse effects of a SAH on the cognitive functioning. We interpret our findings as a decrease in cognitive integrity, which is more affected at baseline as reflected in a heterogeneous profile. In the long term patients recover, however a substantial part of the SAH survivors with seemingly good outcome, present with persistent cognitive impairments. The Rey-Osterrieth complex figure copy appeared to be able to significantly predict cognitive integrity even at a subtle level of dysfunction. From earlier research we know that the majority of the SAH patients experience cognitive complaints in the long term affecting their quality of life [1]. We suggest that these cognitive complaints might reflect the experienced decrease in cognitive integrity as can be predicted by neuropsychological evaluation within three months post SAH.

### References

[1] T.R. Al-Khindi, L. Macdonald and T.A. Schweizer, Cognitive and Functional Outcome After Aneurysmal Subarachnoid Hemorrhage, *Stroke* **41** (2010), 519–536.

[2] H. Blake, M. McKinney, K. Treece, E. Lee and N.B. Lincoln, An evaluation of screening measures for cognitive impairment after stroke, *Age and Ageing* **31** (2002), 451–456.

[3] M.L. Hackett, M.A. Hons and C.S. Anderson, Health outcomes 1 year after subarachnoid hemorrhage, *Neurology* **55** (2000), 658–662.

[4] M.D. Lezak, D.B. Howieson and D.W. Loring, *Neuropsychological Assessment*, (4th ed.), New York: Oxford University Press (2004).

[5] W.T. Longstret, L.M. Nelson, T.D. Koepsell and G. van Belle, Clinical course of spontaneous subarachnoid hemorrhage: a population-based study in King County, Washington, *Neurology* **43** (1993), 712–718.

[6] S.A. Mayer, K.T. Kreiter, M.A. Copeland, G.L. Bernardini, J.E. Bates, S. Peery, J. Claassen, Y.E. Du and E.S. Connolly, Global and domain- specific cognitive impairment and outcome after subarachnoid hemorrhage, *Neurology* **59** (2002), 1750–1758.

[7] J.A. Odgen, E.W.M.B. Mee and M.M.A. Henning, A prospective study of impairment of cognition and memory and recovery after subarachnoid hemorrhage, *Neurosurgery* **33** (1993), 572–587.

[8] P.E.C.A. Passier, J.M.A. Visser-Meily, M.J.E. van Zandvoort, M.W.M. Post, G.J.E. Rinkel and C. van Heugten, Prevalence and determinants of cognitive complaints after aneurismal subarachnoid hemorrhage, *Cerebrovascular Diseases* **29** (2010), 557–563.

[9] R.B. Scott, F. Eccles, A.J. Molyneux, R.S.C. Kerr, P.M. Rothwell and K. Carpenter, Improved Cognitive Outcomes With Endovascular Coiling of Ruptured Intracranial Aneurysms: Neuropsychological Outcomes From the International Subarachnoid Aneurysm Trial (ISAT), *Stroke* **41** (2010), 1743–1747.

[10] J.I. Suarez, Timing of neuropscyological outcome measures in patients with subarachnoid hemorrhage, *Stroke* **28** (2007), 1724–1725.
